# Prediction of remission among patients with a major depressive disorder based on the resting-state functional connectivity of emotion regulation networks

**DOI:** 10.1038/s41398-022-02152-0

**Published:** 2022-09-17

**Authors:** Hang Wu, Rui Liu, Jingjing Zhou, Lei Feng, Yun Wang, Xiongying Chen, Zhifang Zhang, Jian Cui, Yuan Zhou, Gang Wang

**Affiliations:** 1 The National Clinical Research Center for Mental Disorders & Beijing Key Laboratory of Mental Disorders, Beijing Anding Hospital & the Advanced Innovation Center for Human Brain Protection, Capital Medical University, Beijing, China; 2 CAS Key Laboratory of Behavioral Science, Institute of Psychology, Beijing, China; 3 Department of Psychology, University of Chinese Academy of Sciences, Beijing, China

**Keywords:** Predictive markers, Depression

## Abstract

The prediction of antidepressant response is critical for psychiatrists to select the initial antidepressant drug for patients with major depressive disorders (MDD). The implicated brain networks supporting emotion regulation (ER) are critical in the pathophysiology of MDD and the prediction of antidepressant response. Therefore, the primary aim of the current study was to identify the neuroimaging biomarkers for the prediction of remission in patients with MDD based on the resting-state functional connectivity (rsFC) of the ER networks. A total of 81 unmedicated adult MDD patients were investigated and they underwent resting-state functional magnetic resonance imagining (fMRI) scans. The patients were treated with escitalopram for 12 weeks. The 17-item Hamilton depression rating scale was used for assessing remission. The 36 seed regions from predefined ER networks were selected and the rsFC matrix was caculated for each participant. The support vector machine algorithm was employed to construct prediction model, which separated the patients with remission from those with non-remission. And leave-one-out cross-validation and the area under the curve (AUC) of the receiver operating characteristic were used for evaluating the performance of the model. The accuracy of the prediction model was 82.08% (sensitivity = 71.43%, specificity = 89.74%, AUC = 0.86). The rsFC between the left medial superior frontal gyrus and the right inferior frontal gyrus as well as the precuneus were the features with the highest discrimination ability in predicting remission from escitalopram among the MDD patients. Results from our study demonstrated that rsFC of the ER brain networks are potential predictors for the response of antidepressant drugs. The trial name: appropriate technology study of MDD diagnosis and treatment based on objective indicators and measurement. URL: http://www.chictr.org.cn/showproj.aspx?proj=21377. Registration number: ChiCTR-OOC-17012566.

## Introduction

The main characteristics of major depressive disorder (MDD) are depressed mood and anhedonia [1]. As of 2010, it is the second leading factor in influencing years lived with disability among various diseases [2]. Antidepressant medication, such as selective serotonin reuptake inhibitor (SSRI), is the first-line treatment for MDD patients [3]. However, antidepressants are not effective for all MDD patients. In clinical practice, remission is considered the desired outcome, which indicates patients are symptom-free and recovered for the moment [4]. The Sequenced Treatment Alternatives to Relieve Depression (STAR*D) study showed that the overall remission rate was 28% after the first level of treatment [5]. A naturalistic prospective study reported that the remission rate was 43.3% after the acute antidepressant treatment phase, which is usually set to 12 weeks after initiation of treatment [6]. Inadequate antidepressant treatment might prolong the suffering of the patients, and increase the waste of medical resources. Prediction of the patient’s response to drugs based on baseline data might help psychiatrists determine whether a specific drug is suitable for the patient. With the low predictive value of clinical and sociodemographic variables, substantial attention has been directed at identifying neuroimage biomarkers that predict response of antidepressant drugs in MDD patients [7–9].

Multiple neuroimage studies have reported that structural and functional imaging is a potential antidepressant treatment response predictor [10–22] (Table S1). For example, gray matter volume was used to predict clinical remission after 8-week treatment of fluoxetine among 18 MDD patients [11]. The accuracy reported was 88.9%, and the right rostral anterior cingulate cortex, left posterior cingulate cortex, left middle frontal gyrus, and right occipital cortex was identified as predictors of clinical remission. Another study reported 75% accuracy using diffusion MRI to identify the neuroimaging biomarkers which might help in predicting if patients respond to 2 weeks of antidepressant treatment among 85 MDD patients and found that the most sensitive biomarkers for identifying SSRI-improvers were the right hippocampus, left amygdala, right parahippocampal gyrus, right anterior cingulate gyrus, left dorsolateral part of superior frontal gyrus, and left inferior temporal gyrus [22]. More studies apply resting-state functional connectivity (rsFC) to predict antidepressant treatment among MDD patients. An ensemble learning model based on rsFC was designed to predict response to 2 weeks of antidepressants in 98 MDD patients [20]. The obtained accuracy was 80.6% and the bilateral hippocampus, left orbital part superior frontal gyrus, right posterior cingulate gyrus, right amygdala, and left paracingulate gyri were identified as important predictors. In a research study using an index characterizing the reconfiguration of dynamic brain networks, the anterior cingulate cortex (ACC) was reported as a predictor to separate responders from non-responders after antidepressant treatment with escitalopram (≤8 weeks) in a multicenter sample with 106 first-episode MDD patients [21]. An accuracy of 70–89% was achieved by using fMRI to predict whether MDD patients were in remission after 12 weeks of antidepressant treatment [14, 15, 18]. It was found consistently that the prefrontal cortex and the cingulate cortex were important for the prediction of antidepressant efficacy. This suggested that baseline brain image can be used for predicting the response of patients after taking antidepressants, which is of great value in the clinical management of patients with depression. However, there are still some limitations in these studies. First, the follow-up time of studies with a large sample size is relatively short, usually, 2 weeks or 8 weeks [16, 21, 22], which is insufficient to determine whether patients can achieve remission [6]. Second, the sample size of studies with a long follow-up time (12 weeks) is often small [14, 18], which is a major defect for the machine learning model [23].

**Fig. 1:**
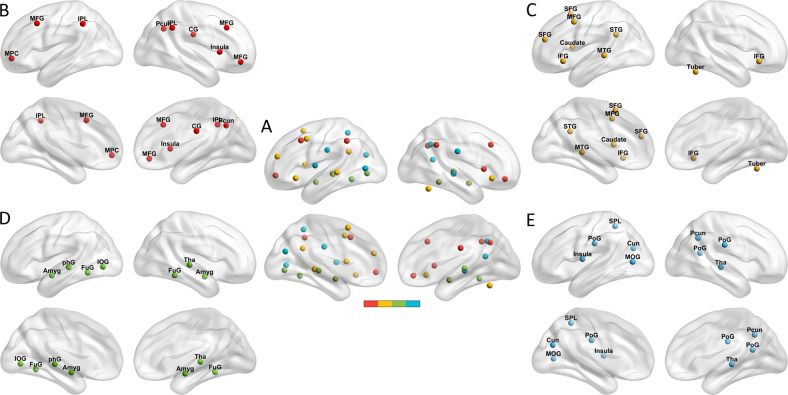
Thirty-six ROIs in the four ER networks. **A** Thirty-six ROIs in the four ER networks. The red, orange, green, and blue nodes corresponded to the first, second, third, and fourth ER network, respectively. **B**–**E** The ROIs of ER1-ER4 were separately displayed. Abbreviations: please see also Table S2.

Although the data used in the above-mentioned studies were obtained from different neuroimaging modalities, it is worthy to be noted that the brain regions that were reported as the main contributors to the prediction of treatment response locate in large-scale brain networks underlying emotion generation, perception, and regulation, which have been identified in a meta-analysis of emotion regulation (ER) [24] (Fig. 1 and Table S2). Based on this meta-analysis, four ER networks were identified, including two cortical networks mainly responding to ER, one subcortical network mainly responsible for emotion perception and generation, and one network implicated in both emotion regulatory processes and emotional reactivity.

Depression has been related to abnormal communication between large-scale brain networks, which can be expressed by rsFC [25]. The predictive effect of this abnormal communication pattern on efficacy prediction has been confirmed by literature studies [16, 20, 21]. The relevant networks of ER were disturbed in MDD patients and the rsFC of related regions were correlated with the severity of depression [26]. Therefore, focusing on the ER networks will help to determine whether it occupies a core position in efficacy prediction, not just overlap in different studies. This can lead to the identification of a unified biomarker for the diagnosis and prognosis of depression. Meanwhile, focusing on the rsFC of the ER networks, the features can be limited to a very small range and thus enable the application of multivariate methods, such as recursive feature elimination [27] and least absolute shrinkage and selection operator [28], which can retain the interaction information of features as much as possible, and enable better prediction performance compared with the method of deleting features by univariate statistics [29].

While the majority of previous studies exploring biomarkers for antidepressant response prediction used a small sample size for monotherapy [11, 17, 30] or mixed antidepressant treatments [12, 19, 20], which might affect the performance of the prediction model, a larger sample size of patients with MDD was recruited in the present study. All patients were unmedicated before recruitment and then were treated with escitalopram for 12 weeks. We postulated that rsFC between ER networks would be useful in predicting whether a MDD patient will obtain remission after 12 weeks of treatment with antidepressants.

## Methods

### Participants

A total of 81 MDD patients were recruited from the Outpatient Department of Beijing Anding Hospital, Capital Medical University, between June 2018 and December 2019. A part of the data (*n* = 40) were used previously and thus clinical assessments, inclusion/exclusion criteria, and diagnostic procedures are provided in the previous study [31]. In short, the patients were diagnosed by experienced psychiatrists with the Mini International Neurological Interview (MINI) 5.0 based on the DSM-IV criteria [32]. All patients were at the time experiencing an episode of depression and were drug free or had taken antidepressants for less than seven days in the last two weeks, and they prepared to use escitalopram. The inclusion criteria also included total score of the 16-Item Quick Inventory of Depressive Symptomatology and Self-Report (QIDS-SR16) ≥ 11 and a score ≥ 14 on the 17-item Hamilton Depression Rating Scale (HAMD-17). Exclusion criteria included any history of bipolar disorder, schizophrenia, schizoaffective disorder, drug and alcohol dependence or acute intoxication or other psychotic disorders; pregnancy or lactation; significant risk of suicidal behavior and HAMD-17 Item 3 (suicide) score ≥ 3; previously intolerance or lack of response to escitalopram and any MRI contraindications; and current clinically significant disease. The study was approved by the Human Research and Ethics Committee of Beijing Anding Hospital, Capital Medical University. Signed informed consent was acquired from all patients.

### Treatment and clinical measurements

Previous studies demonstrated that the remission rate could be observed at 8 weeks or 12 weeks. However, compared with 12 weeks of treatment, shorter (<8 weeks) treatment might be related to a higher risk of developing relapse [33]. Meanwhile, the remission rate increased during 12 weeks of treatment [34]. Therefore, all enrolled patients received a 12-week escitalopram treatment, with the dose increasing from 5 mg/day to 10–20 mg/day according to the condition of each patient. The use of other drugs was not allowed unless the patients experienced insomnia, where they were allowed to use estazolam, lorazepam, or oxazepan. The severity of depression symptoms was evaluated using HAMD-17 in the baseline and 12 weeks. Remission was defined as the HAMD-17 scores ≤ 7 after 12-week treatment [35]. Amongst the MDD patients, 9 patients dropped out during the follow-up period. A total of 72 patients completed the clinical assessment and 12 weeks treatment with antidepressants.

### Data acquisition

Resting-state fMRI scanning was performed using a Siemens Prisma 3.0 T MRI scanner. The structural images were acquired using the T1-weighted magnetization-prepared rapidly acquired gradient-echo (MPRAGE) sequence with the following parameters: TR = 2530 ms; FA = 15°; TE = 1.85 ms; matrix = 256 × 256; FOV = 256 × 256 mm^2^; number of slices = 192; slice thickness = 1 mm; voxel size = 1 × 1 × 1 mm^3^. The resting-state fMRI images were acquired using a gradient-recall echo-planar imaging (GRE-EPI) pulse sequence with the following parameters: TR=2000 ms; TE = 30 ms; FA = 90°; matrix = 64 × 64; FOV = 200 × 200 mm^2^; number of slices = 33; slice thickness = 3.5 mm; gap = 0.7 mm; voxel size = 3.13 × 3.13 × 4.2 mm^3^; phase encoding direction = anterior to posterior; 200 volumes. Patients were instructed to keep their eyes closed, and relax their minds, but not fall asleep during resting-state fMRI scanning. All the patients underwent resting-state fMRI scanning at baseline.

### Data preprocessing

Imaging data were processed using Data Processing & Analysis for (Resting-State) Brain Imaging [36] (DPABI v4.3, http://rfmri.org/DPABI). The processing steps included deletion of the first five volumes, slice timing correction, realignment, segmentation of the T1 images, nuisance variable regression including linear and quadratic trends, the first five principal components of the individually segmented white matter and cerebrospinal fluid, and Friston’s 24 motion parameters, containing six head motion parameters, six head motion parameters one-time point before, and 12 corresponding squared items [37], normalization to the Montreal Neurological Institute (MNI) template, resampling of each voxel to 2 × 2 × 2 mm^3^, spatial smoothing using a 4 mm full-width half-maximum Gaussian kernel and band-pass temporal filtering (0.01–0.1 Hz) [38]. To quantify the impact of head motion, head motion regression with scrubbing was conducted in the preprocessing steps, in which “bad” time points were identified using a threshold of volume-based framewise displacement (FD) (FD > 0.5 mm) [39], as well as one back and two forward neighbors [40]. Subjects who had less than 100 “good” volumes were excluded. Furthermore, the subjects were excluded if the head motion was more than 2.5 mm maximum translation in any direction of *x*, *y*, or *z* or 2.5° of maximum rotation or if their mean FD exceeded three standard deviations of the mean value [31, 41]. Finally, five subjects were removed due to severe motion and a total of 67 MDD patients were included which included 28 remission and 39 non-remission patients.

### Functional connectivity analysis

Inter-regional rsFC analysis was performed using the DPABI software. According to meta-analysis research, 36 regions of interest (ROI) consisted of four ER networks [24]. Each ROI in the ER networks is shown in Fig. 1 (for more details, please see also Table S2 in the supplementary materials). The ER network 1 and ER network 2 contained 10 and 9 ROIs, respectively, responding to ER; the ER network 3 consisted of 8 ROIs, mainly responsible for emotion perception and generation; the ER network 4 included 9 ROIs, mainly involved in emotion stimulation perception in the process of emotion generation and regulation. The ROIs were generated with 5 mm radius spheres based on the peak coordinates of each of the 36 clusters. The time series of voxels of each ROI was extracted and averaged. The rsFC for any pair of two ROIs was calculated by Pearson correlation and then Fisher r-to-Z transformation was conducted. There were (36 × 35)/2 = 630 edges in the low-triangle of the rsFC matrix, which were used as the features for the prediction model.

### Prediction model construction

The construction of prediction model includes two parts: feature selection and model training. A Support Vector Machine based on Recursive Feature Elimination and Cross Validation (SVM-RFECV) algorithm was employed to select the rsFC features [27]. The steps for the feature selection were as follows: (1) the SVM was trained on the training set; (2) ranking criteria were calculated based on the SVM weights and the classification performance through cross-validation; (3) rsFC features were eliminated with the smallest ranking criterion and the classification performance was calculated through cross-validation; (4) steps 3 were repeated until the number of remaining features is equal to the minimum number of features we set. To choose the most discriminative features and obtain stable features, we tested the 100 combinations of the hyperparameter C ranging from [0.1, 0.2, 0.3, …, 1] and the minimum number of features *n* ranging from [10, 20, 30, …100] during SVM–RFECV feature selection. The selected features were then used to train the linear SVM model, and the hyperparameter C’ of this model was adjusted by grid search in a 10-fold cross-validation process. A total of 30 values were extracted equidistantly from the range of 0.01 to 10.

Finally, to improve the accuracy and robustness of the feature selection, Leave-One-Out Cross-Validation (LOOCV) and the area under the curve (AUC) of the Receiver Operating Characteristic (ROC), accuracy, sensitivity, and specificity were used to evaluate the performance of the prediction model. In each LOOCV loop, one subject was set aside to be used as the test set, and the remaining subjects were used as the training set. After 67 loops, each subject had been used as the test set. Moreover, to confirm the important rsFC features in the prediction model, the frequency of each feature was calculated across all the loops, with high frequency indicating higher importance in the ability of remission prediction. The labels corresponding to each patient were randomly disrupted and permutation tests were implemented 1000 times to test the generalization ability of the model. Since the sample size of the non-remission group was larger than that of remission group, this may cause the model prefer to predict patients as non-remission. Therefore, we conducted a bootstrap (randomly selected 20 samples from each of the two groups) 1000 times to test whether the accuracy, sensitivity, and specificity remain stable in a balanced sample set. We also analyzed whether the accuracy of the model could be improved after considering the baseline HAMD-17 item scores. All machine learning analyses were performed using Python and Scikit-learn [42].

### Statistical analysis

We conducted statistical analyses in Statistical Product Service Solutions (SPSS) version 23.0. First, one-sample Kolmogorov–Smirnova test was conducted to estimate the normal distribution of the continuous variables. Two sample t-test or Mann–Whitney U test each was used for analyzing the difference between the remission group and non-remission group for demographic and clinical variables conforming to normal distribution or not. Categorical variables were compared using Chi-square tests. A *p*-value of less than 0.05 was considered to be statistically significant. All tests were two-tailed.

## Results

### Demographic and clinical scales

Table 1 presented the demographic and clinical characteristics of the MDD patients with remission and non-remission, and the remission rate was 41% (28 in remission of 67 patients). Univariate analyses showed that there were no significant differences in age, education level, gender composition, baseline HAMD-17 score, onset frequency of depression, and dosage of escitalopram between the remission patients and the non-remission patients (all *p* > 0.05). Compared with non-remission patients, the remission patients had significantly lower 12-week HAMD-17 scores and a higher percentage of HAMD-17 score reduction (%HAMD-17 = (HAMD-17 scores at baseline - HAMD-17 score at 12 weeks)/HAMD-17 scores at baseline) (all *p* < 0.05). The results of the normality test are demonstrated in Table S3.

**Table 1:** Demographic and clinical characteristics of MDD patients.

	Remission	Non-remission	Z/t/χ^2^	*P* value
	*n* = 28	*N* = 39		
Age (years)	27.34 ± 5.23	26.69 ± 8.34	−1.44	0.147^a^
Education (years)	15.78 ± 2.65	15.48 ± 2.27	−0.69	0.488^a^
Gender (male/female)	10/18	10/29	0.79	0.374^c^
Baseline HAMD-17 score	20.78 ± 3.42	21.82 ± 3.67	−1.24	0.213^a^
12-week HAMD-17 score	4.53 ± 2.08	12.12 ± 3.40	−6.96	<0.001^a^
%HAMD-17	0.77 ± 0.11	0.43 ± 0.17	9.11	<0.001^b^
MDD duration (years)	2.47 ± 3.62	3.39 ± 6.03	−0.24	0.808^a^
Frequency of onset (times)	1.11 ± 1.52	1.05 ± 1.68	−0.32	0.743^a^
Escitalopram (dosage)	6.25 ± 2.20	5.78 ± 1.84	−0.92	0.356^a^

Differences between groups are calculated by Mann–Whitney U test, two-sample t-test, or Chi-square test, each was represented by a, b and c.

### Prediction model

**Fig. 2:**
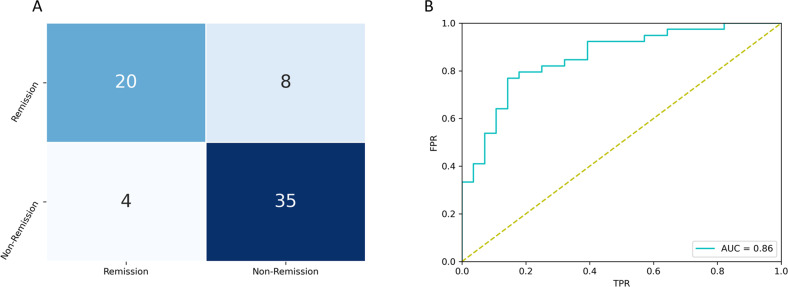
The performance of the prediction model. **A** The confusion matrix of the prediction model. The horizontal ordinate is the predicted value, and the longitudinal ordinate is the actual value. 20 remission group members were correctly predicted as remission, and 35 non-remission group members were correctly predicted as non-remission. **B** The ROC of the prediction model. The AUC is 0.86, accuracy is 82.08% sensitivity is 71.43%, and specificity is 89.74%.

The hyperparameter *C* = 0.1 and the minimum number of features *n* = 40 were selected in the SVM–RFECV. In every loop of LOOCV, a C’ was chosen for the best performance of the model, with the average value being 1.3. The prediction accuracy and performance of SVM are presented in Fig. 2. The AUC of the prediction model was 0.86 (sensitivity = 71.43%, specificity = 89.74%). The accuracy of the prediction model was 82.08% (*p* < 0.001 based on permutation tests). Bootstrap results demonstrated that in balanced small samples, the performance of the model was stable and the mean and standard deviation of accuracy, sensitivity, and specificity were 0.69 ± 0.05, 0.66 ± 0.08, and 0.71 ± 0.07. The addition of the HAMD-17 item scores into the original feature set did not contribute to the improvement of the prediction performance as none of the clinical items was selected for the feature subset.

### Consensus features

**Fig. 3:**
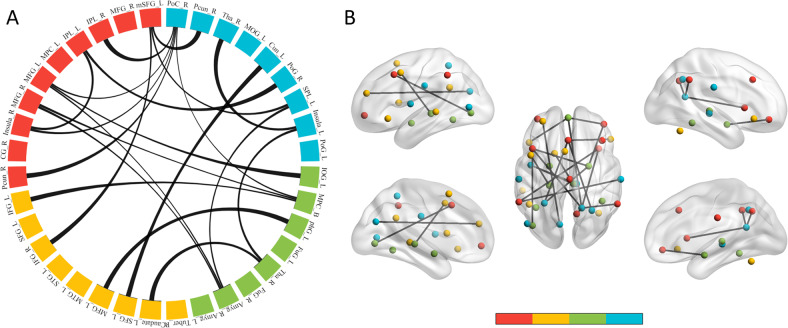
Important features in the prediction model. There were 21 edges, which were selected in at least 90% of the loops (frequency ≥ 61). The red, orange, green, and blue nodes corresponded to the first, second, third, and fourth ER network, respectively. **A** The consensus features were showed in chord picture. **B** The consensus features were showed in brain map. Abbreviations: please see also Table 2.

A total of 176 rsFC features were selected at least once in 67 loops. Among them, the rsFC between the left medial superior frontal gyrus (mSFG, BA8) and the right inferior frontal gyrus (IFG, BA47) and the rsFC between the left mSFG (BA8) and the right precuneus (BA19) were selected in every loop. A total of 21 edges were selected in at least 90% of the cycles (frequency ≥ 61), indicating these features have a high prediction ability of the treatment response (Fig. 3). The majority of the edges were the rsFC between different ER networks and mainly belonged to the rsFC between ER network 1 and other ER networks, especially with the subcortical ER network (i.e., ER network 3) and the intermediatory network (i.e., ER network 4) (Table 2).

**Table 2:** Consensus features in the prediction model.

Brain region	ER	BA	Brain region	ER	BA	*f*
Left medial superior frontal gyrus	1	8	Right inferior frontal gyrus	2	47	67
			Right precuneus	4	19	67
			Left superior parietal lobule	4	7	66
Right inferior parietal lobule	1	40	Right posterior cingulate	4	30	64
Left inferior parietal lobule	1	40	Right postcentral gyrus	4	2	64
			Right insula	1	13	64
Left middle frontal gyrus	1	6	Right amygdala	3	*	62
			Left inferior occipital gyrus	3	19	62
			Right posterior cingulate	4	30	61
Right middle frontal gyrus	1	11	Right amygdala	3	*	61
			Bilateral medial prefrontal cortex	3	10	61
Right insula	1	13	Bilateral medial prefrontal cortex	3	10	61
Right precuneus	1	7	Right posterior cingulate	4	30	66
Left inferior frontal gyrus	2	47	Bilateral medial prefrontal cortex	3	10	66
Left middle frontal gyrus	2	6	Left parahippocampal gyrus	3	27	66
Left superior frontal gyrus	2	9	Left cuneus	4	18	66
Left caudate	2	*	Right thalamus	3	*	66
Right amygdala	3	*	Left superior parietal lobule	4	7	65
Right thalamus	3	*	Left insula	4	13	65
Bilateral medial prefrontal cortex	3	10	Right posterior cingulate	4	30	65
Left insula	4	13	Right thalamus	4	*	64

The ER and BA each represent the ER network or Brodmann area to which the ROI belongs; *f* denotes the frequency of the edge selected as important features in total of 67 loops; * indicates the regions out of Brodmann areas.

## Discussion

The predictive potential of rsFC in ER networks at baseline was investigated in patients after a 12-week escitalopram treatment in this study. The accuracy of the prediction model was 82.08%, which points that rsFC may be a clinically applicable predictor, for individual discrimination between patients likely to remit from depressive episodic and patients who are not, after a 12-week escitalopram treatment regime. Specifically, results showed that the rsFC in brain networks supporting ER predicts clinical remission following antidepressant treatment. Additionally, the rsFC between the left mSFG and the right inferior frontal gyrus as well as the precuneus were the features with the highest discriminative ability in predicting remission from escitalopram among MDD patients.

The current study focused on the rsFC in the ER networks, which is different from previous studies exploring biomarkers for antidepressant effect prediction. The majority of previous studies [16, 17, 19, 43] focused on feature investigation at a whole brain level based on univariate statistics, which may be affected by the noise signals and ignore the features that may not be statistically significant but may contain important information when interacting with other features [44, 45]. In addition, by focusing on specific networks, the features to be filtered were limited to a very small range, thus RFECV could be used for filtering, which can keep the interaction information of features as much as possible compared with the method of deleting features by univariate statistics [29]. The prediction performance of the proposed model (AUC = 0.86, sensitivity = 71.43%, specificity = 89.74%) obtained in the current study was higher compared with other literature studies [17, 18, 21, 30]. At the same time, another group explored the relationship between the dynamic functional connectivity (dFC) based on the ER networks and the efficacy of antidepressants. They demonstrated that the predicted reduction rate of HAMD scores based on the strength of baseline dFC was significantly correlated with the actual reduction rate of HAMD scores. This finding also illustrated the role of ER networks in predicting the efficacy of antidepressants [46]. The above finding and our study suggest a direction in which future studies could focus, namely on the features with potential prediction efficacy found by previous studies, rather than screening features in the whole brain range by univariate statistics.

Out of the total 630 features that were investigated, only 21 were selected as the most predictive and stable features, which further supports the assumption of Yamashita [47] that mental illness is due to the dysfunction of a part of the rsFC of the brain, rather than the whole brain. The most important features reported in this study were the rsFC between the ER network 1 and other ER networks, especially with the subcortical ER network (ER network 3) and the intermediatory network (ER network 4). ER network 1 is responsible for voluntary emotion regulation, ER network 3 is associated with emotion perception and generation, and ER network 4 plays an intermediary role in re-appraisal and integrates information from the prefrontal networks (ER network 1 and 2) as well as the subcortical network (ER network 3) to generate emotional responses and regulate these response processes as well as emotional reactivity [24]. Our finding suggests that functional interaction between the regions responsible for a higher level of ER and the regions involved in the lower levels of regulation is vital for the prediction of treatment outcomes in MDD. The rsFC between the left mSFG and the right IFG and between the left mSFG and the precuneus were considered as the features with the highest discrimination ability in this model because they were selected in every loop of LOOCV. The left mSFG, a part of in dorsomedial prefrontal cortex (dmPFC), is involved in voluntary emotion regulation with other regions in ER network 1 [24]. In previous studies, a region near the left mSFG in our study, which is often called ACC, was also suggested to be critical in the prediction of treatment efficacy [21, 48, 49].

This study also has some limitations. First, despite the high accuracy achieved in the current study, whether the model can still achieve high accuracy on a dataset with a larger sample size or from multiple sites remains to be verified. However, combined with previous studies, we have reason to believe that this large-scale brain network contains rich efficacy prediction information [46]. Second, the patients in this study used only escitalopram as antidepressants, which ensure the homogeneity of our sample, but in the real world, the patients used far more kinds of drugs, which makes it more difficult for the model to accurately predict. Third, the current study only focused on the functional profile of the ER networks (i.e., rsFC). Previous studies have suggested that structural features of brain regions in the ER networks also possess the predictive ability of treatment responses. Structural and functional features can be integrated with future studies to assess whether a better prediction performance can be achieved. Fourth, the spheres drawn by the peak coordinates as ROI were chosen but not the clusters, which may lead to loss of information in the brain regions. Finally, behavior measurements on ER are lacking in this study. The dysfunction of emotion processing and regulation is one of the prominent features in the pathophysiology of MDD [26, 50]. Thus, it is unknown whether the addition of these behavior measurements into the original feature set can further improve prediction performance. However, the addition of HAMD-17 item scores into the original feature set, which may reflect problems in ER, did not improve the prediction performance, as none of the clinical items were selected in the feature subset.

## Conclusions

In conclusion, by focusing on the rsFC in the emotion regulation networks, we demonstrated that using features from this rsFC can predict the performance of remission in patients with MDD. These findings suggested that the rsFC of emotion regulation networks has the potential to be used as a biomarker for predicting the treatment response of MDD patients to escitalopram. Future studies can test the generalization ability of this prediction model using larger sample sizes and/or in multi-center datasets and explore whether the relevant rsFC will be changed with the progress of treatment.

## Supplementary information

Supplemental Material

## Data Availability

The code that support the findings of this study are available from the corresponding authors upon reasonable request.
