# Supplementary material for: Prediction of remission among patients with a major depressive disorder based on the resting-state functional connectivity of emotion regulation networks
Source: Transl Psychiatry. 2022 Sep 17;12:391. doi: 10.1038/s41398-022-02152-0 (PMC9482642; doi:10.1038/s41398-022-02152-0)
Supplement: Supplementary file 1 — Supplemental Material [file 41398_2022_2152_MOESM1_ESM.doc]

**Prediction of remission among patients with a major depressive disorder based on the resting-state functional connectivity of emotion regulation networks**

**Supplemental Material**

**Table S1. A summary of prediction models on antidepressant efficiency.**

| Studies | Sample Size | Intervention | Duration | Modality | Method | Brain Region | Accuracy |
| --- | --- | --- | --- | --- | --- | --- | --- |
| Marquand et al. (2008) | 9 | SSRI | 8 weeks | fMRI | SVM | anterior cingulate gyrus, Middle frontal gyrus, Inferior temporal gyrus, Cerebellum, Superior frontal gyrus, Superior temporal gyrus, Middle temporal gyrus, Precuneus, Lingual gyrus, Midbrain | 67% |
| Costafreda et al. (2009) | 18 | SSRI or CBT | 8 weeks | sMRI | SVM | anterior and posterior cingulate cortices, orbitofrontal cortex | 88.9% |
| Nouretdinov et al. (2011) | 18 | antidepressant medication | 8 weeks | sMRI | transductive conformal predictor | anterior and posterior cingulate cortices, orbitofrontal cortex | 77%/83% |
| Gong et al. (2011) | 46 | SSRI or SNRI | 6 weeks | sMRI,DTI | SVM | Right middle frontal, Left inferior parietal, Right supramarginal gyrus, Left paracentral lobule, Right middle temporal et al. | 69%/65% |
| Patel et al. (2015) | 33 | SSRI or SNRI | 12 weeks | fMRI,sMRI,DTI | SVM,  linear regression,alternating decision tree | medial prefrontal cortex, anterior and posterior cingulate cortex, thalamus, hippocampus, angular gyrus, orbitofrontal cortex, superior frontal gyrus, precuneus, midcingulate cortex | 89% |
| Dunlop et al. (2017) | 122 | SSRI or SNRI or CBT | 12 weeks | fMRI | 2×2 analysis of variance | subcallosal cingulate cortex，anterior ventrolateral prefrontal cortex, insula, dorsal midbrain, ventromedial prefrontal cortex | 72%-78% |
| Karim et al. (2018) | 49 | SNRI | 12-weeks | fMRI | logistic classification | frontal cortex, hippocampus, parahippocampus, caudate,thalamus, medial temporal cortex, middle cingulate | Sensitivity=72% ，specificity=68% |
| Goldstein-Piekarski et al. (2018) | 80 | SSRI or SNRI | 8 weeks | fMRI | logistic regression | posterior cingulate cortex,anterior cingulate cortex, medial prefrontal cortex | 81% |
| Godlewska et al. (2018) | 32 | SSRI | 6 weeks | fMRI | cut-off value | anterior cingulate cortex | 71% |
| Tian et al. (2020) | 106 | SSRI | >2 weeks | fMRI | SVM | anterior cingulate cortex | 79% |
| Pei et al. (2020) | 98 | SSRI or SNRI | 2 weeks | fMRI | SVM | hippocampus, superior frontal gyrus, posterior cingulate gyrus, amygdala,paracingulate gyri | 81% |
| Korgaonkar et al. (2020) | 163 | SSRI or SNRI | 8 weeks | fMRI | ANOVA | default mode network, frontoparietal, somatomotor networks | 61%/ 68% |
| Xue et al. (2021) | 118 | SSRI or SNRI | 2-weeks | DTI | SVM | hippocampus, amygdala, putamen,superiorfrontal gyrus | 77% |

**Table S2. The characteristics of 36 ROIs in emotional regulation networks.**

| ER networks | SIDE | Brain Region | Abbreviation | BA | Peak Coordinates | | |
| --- | --- | --- | --- | --- | --- | --- | --- |
|  |  |  |  |  | x | y | z |
| ER 1 | L | Medial Superior Frontal Gyrus | mSFG_L | 8 | 0 | 24 | 50 |
|  | R | Middle Frontal Gyrus | MFG_R | 8 | 40 | 24 | 42 |
|  | R | Inferior Parietal Lobule | IPL_R | 40 | 58 | −52 | 38 |
|  | L | Inferior Parietal Lobule | IPL_L | 40 | −58 | −50 | 44 |
|  | L | Medial Prefrontal cortex | MPC_L | 10 | −36 | 52 | −2 |
|  | L | Middle Frontal Gyrus | MFG_L | 6 | −42 | 14 | 48 |
|  | R | Middle Frontal Gyrus | MFG_R | 11 | 42 | 46 | −8 |
|  | R | Insula | Insula_R | 13 | 36 | 16 | 6 |
|  | R | Cingulate Gyrus | CG_R | 23 | 2 | −22 | 30 |
|  | R | Precuneus | Pcun_R | 7 | 10 | −64 | 36 |
| ER 2 | L | Inferior Frontal Gyrus | IFG_L | 47 | −46 | 24 | −8 |
|  | L | Superior Frontal Gyrus | SFG_L | 6 | −4 | 10 | 62 |
|  | R | Inferior Frontal Gyrus | IFG_R | 47 | 50 | 28 | −8 |
|  | L | Superior Temporal Gyrus | STG_L | 39 | −46 | −52 | 28 |
|  | L | Middle Temporal Gyrus | MTG_L | * | −54 | −34 | −2 |
|  | L | Middle Frontal Gyrus | MFG_L | 6 | −44 | 6 | 50 |
|  | L | Superior Frontal Gyrus | SFG_L | 9 | −30 | 48 | 26 |
|  | L | Caudate | Caudate_L | * | −16 | 10 | 12 |
|  | R | Tuber | Tuber_R | * | 36 | −60 | −30 |
| ER 3 | L | Amygdala | Amyg_L | * | −22 | −4 | −16 |
|  | R | Amygdala | Amyg_R | * | 24 | −4 | −18 |
|  | R | Fusiform Gyrus | FuG_R | 37 | 40 | −46 | −18 |
|  | R | Thalamus | Tha_R | * | 6 | −26 | 0 |
|  | L | Fusiform Gyrus | FuG_L | 37 | −38 | −54 | −14 |
|  | L | Parahippocampal Gyrus | phG_L | 27 | −22 | −28 | −4 |
|  | B | Medial Prefrontal cortex | MPC_B | 10 | 0 | 54 | −10 |
|  | L | Inferior Occipital Gyrus | IOG_L | 19 | −42 | −76 | −6 |
| ER 4 | L | Postcentral Gyrus | PoG_L | 2 | −58 | −22 | 32 |
|  | L | Insula | Insula_L | 13 | −44 | −4 | 10 |
|  | L | Superior Parietal Lobule | SPL_L | 7 | −28 | −52 | 56 |
|  | R | Postcentral Gyrus | PoG_R | 2 | 62 | −22 | 30 |
|  | L | Cuneus | Cun_L | 18 | −10 | −76 | 22 |
|  | L | Middle Occipital Gyrus | MOG_L | 19 | −48 | −74 | 2 |
|  | R | Thalamus | Tha_R | * | 10 | −26 | −4 |
|  | R | Precuneus | Pcun_R | 19 | 28 | −60 | 38 |
|  | R | Posterior Cingulate | PoC_R | 30 | 16 | −56 | 16 |

Note: The ER networks’ number represents which ER network the subsequent ROIs belongs to.

**Table S3. Normality test of demographic and clinical characteristics.**

|  | statistic | df | p |
| --- | --- | --- | --- |
| Age (years) | 0.11 | 66 | 0.039 |
| Education (years) | 0.36 | 66 | <0.001 |
| Baseline HAMD-17 score | 0.11 | 66 | 0.028 |
| 12-week HAMD-17 score | 0.12 | 66 | 0.013 |
| %HAMD-17 | 0.10 | 66 | 0.170 |
| MDD duration (years) | 0.28 | 66 | <0.001 |
| Frequency of onset | 0.35 | 66 | <0.001 |
| Escitalopram (dosage) | 0.49 | 66 | <0.001 |

**References**

1. Costafreda, S. G., Chu, C., Ashburner, J., & Fu, C. H. Y. (2009). Prognostic and diagnostic potential of the structural neuroanatomy of depression. *PloS One*, *4*(7), e6353. https://doi.org/10.1371/journal.pone.0006353
2. Dunlop, B. W., Rajendra, J. K., Craighead, W. E., Kelley, M. E., McGrath, C. L., Choi, K. S., Kinkead, B., Nemeroff, C. B., & Mayberg, H. S. (2017). Functional Connectivity of the Subcallosal Cingulate Cortex And Differential Outcomes to Treatment With Cognitive-Behavioral Therapy or Antidepressant Medication for Major Depressive Disorder. *American Journal of Psychiatry*, *174*(6), 533–545. https://doi.org/10.1176/appi.ajp.2016.16050518
3. Godlewska, B. R., Browning, M., Norbury, R., Igoumenou, A., Cowen, P. J., & Harmer, C. J. (2018). Predicting Treatment Response in Depression: The Role of Anterior Cingulate Cortex. *The International Journal of Neuropsychopharmacology*, *21*(11), 988–996. https://doi.org/10.1093/ijnp/pyy069
4. Goldstein-Piekarski, A. N., Staveland, B. R., Ball, T. M., Yesavage, J., Korgaonkar, M. S., & Williams, L. M. (2018). Intrinsic functional connectivity predicts remission on antidepressants: A randomized controlled trial to identify clinically applicable imaging biomarkers. *Translational Psychiatry*, *8*(1), 57. https://doi.org/10.1038/s41398-018-0100-3
5. Gong, Q., Wu, Q., Scarpazza, C., Lui, S., Jia, Z., Marquand, A., Huang, X., McGuire, P., & Mechelli, A. (2011). Prognostic prediction of therapeutic response in depression using high-field MR imaging. *NeuroImage*, *55*(4), 1497–1503. https://doi.org/10.1016/j.neuroimage.2010.11.079
6. Karim, H. T., Wang, M., Andreescu, C., Tudorascu, D., Butters, M. A., Karp, J. F., Reynolds, C. F., & Aizenstein, H. J. (2018). Acute trajectories of neural activation predict remission to pharmacotherapy in late-life depression. *NeuroImage: Clinical*, *19*, 831–839. https://doi.org/10.1016/j.nicl.2018.06.006
7. Korgaonkar, M. S., Goldstein-Piekarski, A. N., Fornito, A., & Williams, L. M. (2020). Intrinsic connectomes are a predictive biomarker of remission in major depressive disorder. *Molecular Psychiatry*, *25*(7), 1537–1549. https://doi.org/10.1038/s41380-019-0574-2
8. Marquand, A. F., Mourão-Miranda, J., Brammer, M. J., Cleare, A. J., & Fu, C. H. Y. (2008). Neuroanatomy of verbal working memory as a diagnostic biomarker for depression. *NeuroReport*, *19*(15), 1507–1511. https://doi.org/10.1097/WNR.0b013e328310425e
9. Nouretdinov, I., Costafreda, S. G., Gammerman, A., Chervonenkis, A., Vovk, V., Vapnik, V., & Fu, C. H. Y. (2011). Machine learning classification with confidence: Application of transductive conformal predictors to MRI-based diagnostic and prognostic markers in depression. *NeuroImage*, *56*(2), 809–813. https://doi.org/10.1016/j.neuroimage.2010.05.023
10. Patel, M. J., Andreescu, C., Price, J. C., Edelman, K. L., Reynolds, C. F., & Aizenstein, H. J. (2015). Machine learning approaches for integrating clinical and imaging features in late-life depression classification and response prediction: Prediction models for late-life depression. *International Journal of Geriatric Psychiatry*, *30*(10), 1056–1067. https://doi.org/10.1002/gps.4262
11. Pei, C., Sun, Y., Zhu, J., Wang, X., Zhang, Y., Zhang, S., Yao, Z., & Lu, Q. (2020). Ensemble Learning for Early-Response Prediction of Antidepressant Treatment in Major Depressive Disorder. *Journal of Magnetic Resonance Imaging: JMRI*, *52*(1), 161–171. https://doi.org/10.1002/jmri.27029
12. Tian, S., Sun, Y., Shao, J., Zhang, S., Mo, Z., Liu, X., Wang, Q., Wang, L., Zhao, P., Chattun, M. R., Yao, Z., Si, T., & Lu, Q. (2020). Predicting escitalopram monotherapy response in depression: The role of anterior cingulate cortex. *Human Brain Mapping*, *41*(5), 1249–1260. https://doi.org/10.1002/hbm.24872
13. Xue, L., Pei, C., Wang, X., Wang, H., Tian, S., Yao, Z., & Lu, Q. (2021). Predicting Neuroimaging Biomarkers for Antidepressant Selection in Early Treatment of Depression. *Journal of Magnetic Resonance Imaging: JMRI*. https://doi.org/10.1002/jmri.27577
